# Treatment of humeral shaft fractures: a new minimally-invasive plate osteosynthesis versus open reduction and internal fixation: a case control study

**DOI:** 10.1186/s12893-021-01347-4

**Published:** 2021-09-23

**Authors:** Jing Yang, Dapeng Liu, Lina Zhang, Zhanxin Lu, Tang Liu, Cheng Tao

**Affiliations:** 1 Department of Orthopedics, The Second Xiangya Hospital, Central South University, Changsha, 410000 Hunan China; 2 Department of Orthopedics, The Fifth Affiliated Hospital of Xinjiang Medical University, Urumqi, 830000 Xinjiang China; 3 Department of Mental Health Institute, The Second Xiangya Hospital, Central South University, Changsha, 410000 Hunan China

**Keywords:** Humeral shaft fractures, MIPO, Anteromedial, Minimally-invasive

## Abstract

**Background:**

To evaluate the feasibility and safety of a new minimally-invasive surgical approach–anteromedial minimally-invasive plate osteosynthesis (MIPO)–in the treatment of middle and distal humeral shaft fractures.

**Methods:**

Fourteen patients with humeral shaft fracture treated with anteromedial MIPO from November 2016 to March 2020 (MIPO Group) were selected as the study subjects. Open reduction and internal fixation (ORIF) were used to treat 14 patients with humeral shaft fractures as the control group (ORIF group). The two groups were fixed with a locking compression plate (LCP) or LCP + multi-directional locking screw system (MDLS). The incision length, intraoperative blood loss, intraoperative fluoroscopy time, operation time, length of hospital stay, fracture healing time, QuickDASH score and Constant score were observed and compared between the two groups.

**Results:**

Fourteen patients were enrolled in each group. The incision length (7.79 ± 2.39 cm), intraoperative blood loss (96.07 ± 14.96 mL), operative time (110.57 ± 21.90 min), hospital stay (6.29 ± 1.49 days) and fracture healing time (14.94 ± 0.99 weeks) in the MIPO group were all lower than those in the ORIF group, and the difference was statistically significant for each parameter (*P* < 0.05). The intraoperative fluoroscopy time (20.07 ± 3.22) in the MIPO group was significantly higher than that in the ORIF group (*P* < 0.05). There were no significant differences in age (*P* = 0.078), QuickDASH score (*P* = 0.074) or Constant score (*P* = 0.293) between the two groups and no postoperative complications occurred in any of the patients.

**Conclusion:**

The anteromedial approach MIPO technique has the advantages of less trauma, less bleeding, low risk of nerve injury and high rate of fracture healing. It is one of the most effective methods for the treatment of middle and middle–distal humeral shaft fractures.

## Background

Humeral shaft fractures account for 2–4% of all fractures [1], yet at present, there is no clear gold standard for the treatment of humeral shaft fracture [2, 3]. Although most humeral shaft fractures can be treated nonoperatively, surgical treatment leads to better fracture reduction and early functional exercise [4]. However, dissection of soft tissue during open reduction can affect the blood supply to the fracture, increasing the risk of fracture nonunion, incision infection, and iatrogenic nerve injury. With the mature application of minimally-invasive plate osteosynthesis (MIPO) in the treatment of fractures, MIPO has been used as an alternative and has achieved good results [5]. Some authors reported using the anterolateral minimally-invasive approach, and found that the incidence of distal incision iatrogenic radial nerve palsy remained high [6]. Iatrogenic injury of the radial nerve is related to its special anatomical location and locus [1]. The purpose of our study was to report our experience in the treatment of middle and distal humerus fractures with an anteromedial approach to MIPO [7]. We aimed to evaluate the feasibility and safety of the surgical approach, and to evaluate the postoperative function of the upper limb [8].

## Methods

### General information

The study was reviewed and approved by the institutional ethics board of the hospital; all patients gave informed consent and agreed to participate in our study. We performed a retrospective analysis of patients treated between November 2016 and February 2020 at our hospital. The medical records of patients with humeral shaft fractures admitted to our hospital were analyzed. Inclusion criteria: (1) patients diagnosed with unilateral closed humeral shaft fractures by imaging examination; (2) no neurovascular damage; (3) the patient consented to surgery. Exclusion criteria: (1) pathological fracture; (2) combined with nerve injury; (3) open fracture; (4) a history of mental illness or cognitive dysfunction; (5) patients with severe organic diseases who would be unable to tolerate the treatment in this study.

The MIPO group comprised eight males and six females, between the ages of 25 and 81 (mean age 47.79 ± 18.61). There were 11 cases on the left side and three cases on the right side. AO type: A1.2:3; A1.3: one case; A3.2: four cases; A2.2: two cases; B1.2: one case; B1.3: three cases. The ORIF group comprised five males and nine females. aged from 16 to 73 years (mean 47.79 ± 18.61). AO type: A3.2: four cases; A1.2: one case; A2.2: one case; A1.3: two cases; B1.3: five cases; A3.3: one case. There were no significant differences between the general characteristics of the two groups (*P* > 0.05).

### Surgical technique

**Fig. 1:**
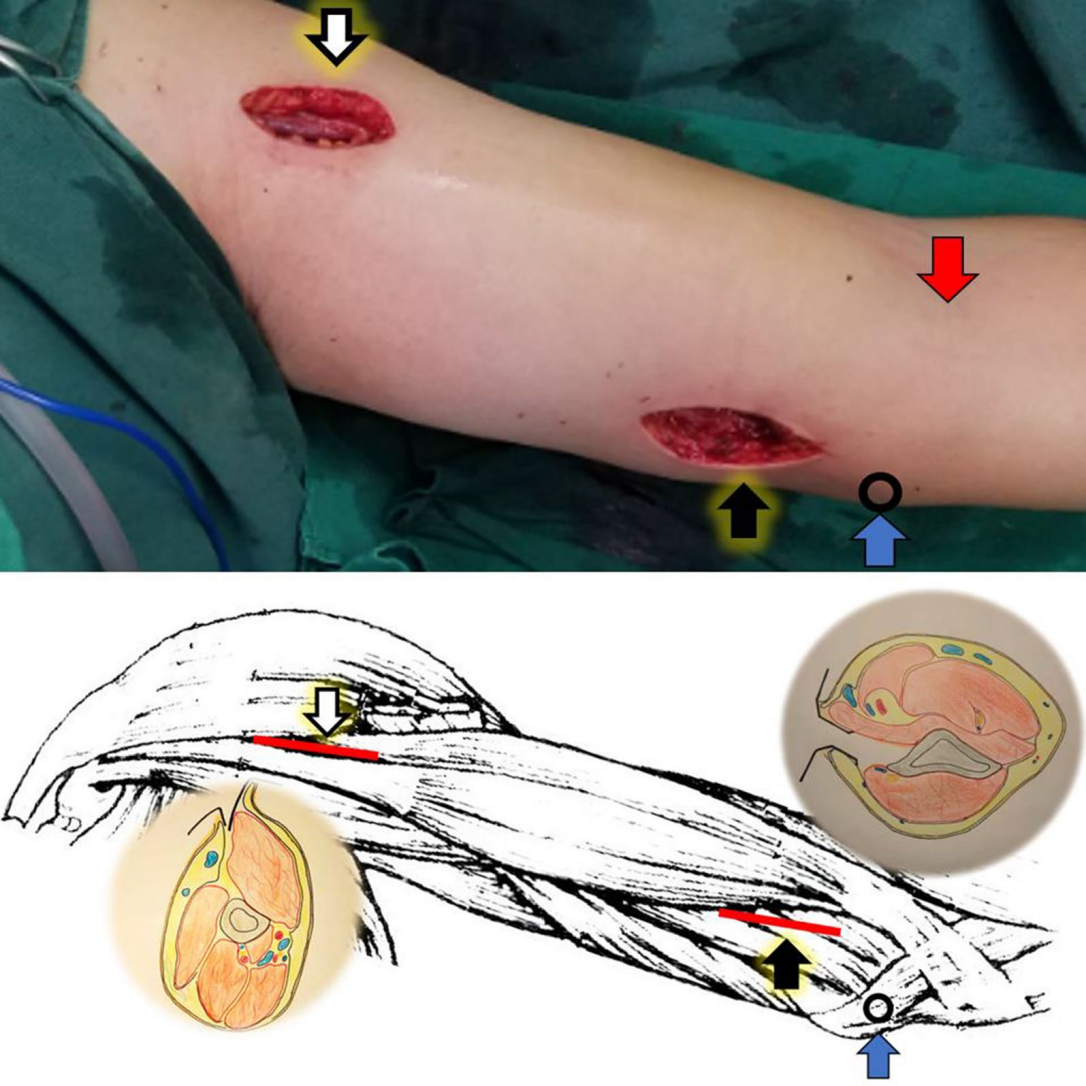
The proximal incision (white arrow in artist’s illustration); the distal incision (black arrow in artist’s illustration) made along the medial margin of the biceps and proximal to the elbow flexion crease (red arrow). Proximal and distal incisions of the left arm diagrams showing the plane of dissection; (blue arrow: the medial epicondyle)

**Fig. 2:**
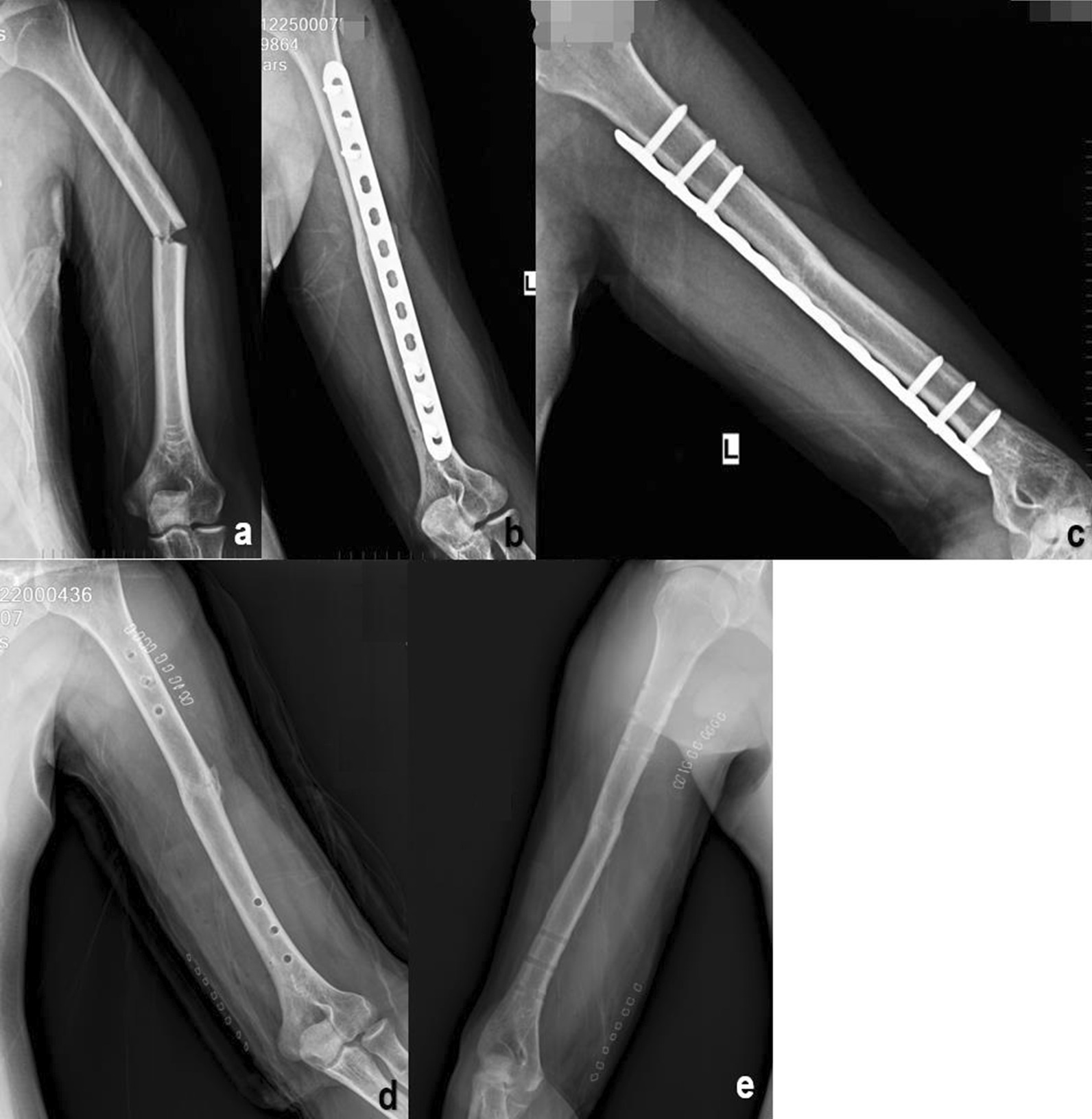
**a** A 38-year-old man (case 3) who was involved in a road traffic accident and sustained a middle fracture of the left humeral shaft. **b**, **c** 12 months after surgery, the bone was clinically united in anatomical alignment. **d**, **e** 24 months after surgery, in accordance with the wishes of the patient, the internal fixation was removed

**Fig. 3:**
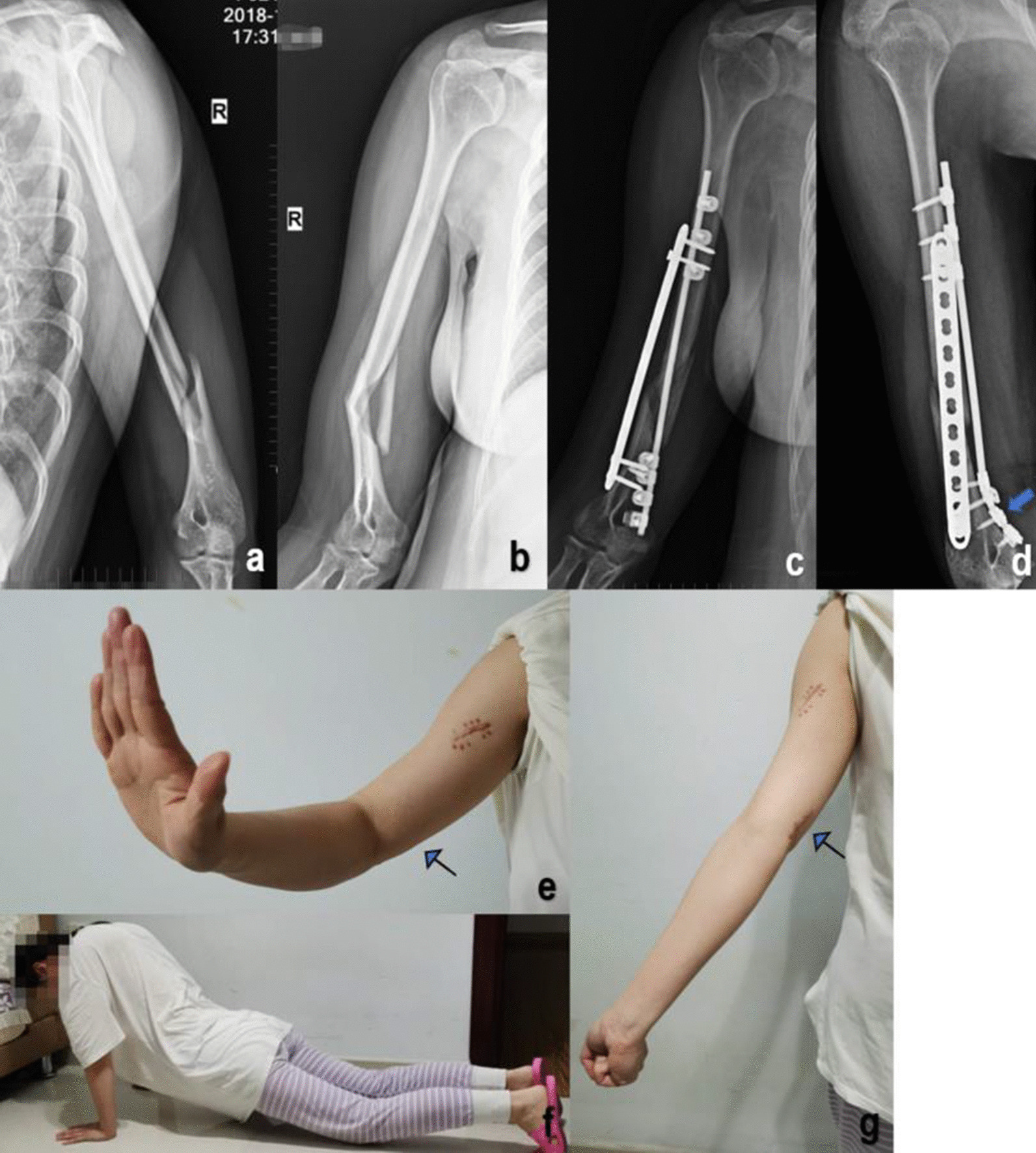
**a**, **b** A Patient number 13 in the MIPO group, a 31-year-old patient who suffered a distal humerus fracture after a fall. AO/OTA: *B1.3.* Preoperative X-rays. **c**, **d** The patient was treated with the anteromedial + anterolateral MIPO technique and fixed with LCP + MDLS (arrow: fixation of a single miniplate with multi-directional locking screw). **e–g** 28 months after surgery, with full recovery of function. (arrow: well-hidden scar at the elbow)

MIPO group: patients were administered brachial plexus nerve tissue anesthesia, then positioned with the trunk supine, the arm and shoulder abducted 90 degrees, and the forearm in complete supination. The medial epicondyle was first palpated and the incision was begun 1 cm in front of the medial epicondyle. To determine the space between the biceps and triceps brachii, 3–4 cm of skin was cut proximally along the biceps groove. The basilar vein and medial forearm cutaneous nerve were identified and protected, the brachialis muscle fascia was incised, and the anteromedial surface of the distal humerus was exposed. The LCP was placed on the skin to determine the location of the proximal humerus incision, and the proximal incision was determined by palpating the space between the proximal biceps and the medial margin of the deltoid (Fig. 1). After determining the insertion point of the pectoralis major tendon, the long head tendon of the biceps brachii was pulled medially or lateral and the dissection was continued downward to the medial surface of the proximal shaft of the humerus. To achieve full exposure, part of the pectoralis major insertion could be removed and subcutaneous MIPO tunnels created, connecting the distal and proximal incisions. The steel plate was inserted from the distal end to the proximal, and the position of the steel plate was adjusted by locking the drill bushings at the distal and proximal ends. The fracture was then reduced with the aid of fluoroscopy. Once the reduction was satisfactory, a lag screw was drilled proximally to help position the fracture reduction, the shoulder and elbow were moved, no impact was confirmed, and the proximal and distal locking screws were drilled sequentially, using at least three proximal screws. If the distal end was near the medial condyle, a single cortical locking screw was selected for fixation, and the incision was sutured without an indwelling drainage tube. A typical case is shown in Fig. 2. In this group, three patients (A3.2, B1.3, A1.3) had a distal 1/3 humerus fracture. Because of the particularity of the fracture fragment and the distal humerus, in order to ensure the fracture had excellent stability and promote early functional exercise for patients after surgery, the lateral minimally-invasive plate bracing technique was required to achieve lateral and medial bracing and cross screw fixation [9]. In this study, we selected the anteromedial + anterolateral MIPO technique and fixation with LCP + MDLS for the patients with distal humerus fractures. A typical case is shown in Fig. 3.

ORIF group patients received brachial plexus nerve tissue anesthesia, then patients were placed in the supine position (10 cases) or the prone position (four cases), and open reduction and plate internal fixation were performed by conventional anterolateral and posterior approaches centering on the fracture site. Patients were placed in the supine or prone position, and their arms were placed on a radiologically-transparent plate. In both approaches, the radial nerve was exposed, the fracture site was exposed, hematoma and soft tissue between the fragments were removed, and the fracture was reduced. The anterolateral incision approach was used to fix the fracture with the LCP. In the posterior approach group, a double LCP was placed medially and laterally on the humerus. Passive movement of the shoulder and elbow joints was then used to examine the stability of the bone plate structure, a drainage tube was placed under the muscle, then the incision was sutured [10].

### Postoperative management

Postoperatively, a forearm sling was used for 2 weeks, and shoulder and elbow joints were passively moved. After 2 weeks, the shoulder and elbow joints were gradually moved actively. After X-ray imaging showed the presence of a bone connection at the fracture end, strength exercises were performed. Patients with radial nerve injury would be given drugs to promote nerve recovery, but none of the patients included in this study had radial nerve injury. X-ray examination was performed 3 days after surgery, and outpatient examination was performed 1 and 3 months after surgery. X-ray examination was performed every 6 months thereafter to observe fracture healing. QuickDASH score and Constant score were given at the last follow-up to evaluate the postoperative recovery effect.

### Observation indicators

MIPO Group (Table 1), ORIF Group (Table 2). Incision length (cm), intraoperative blood loss (mL), intraoperative X-ray fluoroscopy (times), operation time (minutes), hospital stay (days), fracture healing time (months), follow-up time (months), QuickDASH score and Constant score were all evaluated and compared.

**Table 1:** MIPO group

Case	Age (years)	Gender	Side	AO/OTA classification	Plate type	Incision length (cm)	Bleeding volume (ml)	Fluoroscopy times	Operative time (min)	HSD (days)	BH (weeks)	FFU (months)	Post-op RNP	QuickDASH score	Constant score
1	63	Male	Left	A1.2	LCP	6	90	22	110	8	15.2	12	NA	0.0	90
2	46	Female	Left	A1.2	LCP	8	100	18	98	5	14.31	13	NA	4.5	85
3	38	Male	Left	A3.2	LCP	7	95	19	90	4	14.2	29	NA	2.3	86
4	51	Male	Left	A1.2	LCP	6	110	27	95	5	15.3	34	NA	2.3	90
5	45	Male	Left	A3.2	LCP	6	85	15	105	6	16.2	15	NA	3.2	80
6	26	Male	Left	A3.2	LCP + MDLS	10	80	16	140	5	15.3	12	NA	2.3	86
7	64	Male	Left	A2.2	LCP	6	80	20	100	7	14.3	12	NA	0.0	100
8	63	Female	Left	A2.2	LCP	6	90	25	113	6	15.4	14	NA	3.2	88
9	74	Female	Right	B1.2	LCP	7	110	19	105	8	16.4	12	NA	2.2	90
10	25	Female	Right	B1.3	LCP	8	95	21	95	5	15.3	14	NA	4.5	80
11	35	Female	Left	B1.3	LCP	8	75	19	90	6	14	13	NA	3.5	90
12	81	Male	Left	A3.2	LCP	6	110	18	102	8	16.2	12	NA	3.2	85
13	31	Female	Right	B1.3	LCP + MDLS	11	95	20	145	6	13.0	28	NA	2.2	95
14	27	Male	Left	A1.3	LCP + MDLS	14	130	22	160	9	14	36	NA	2.0	95

*BH* bone healing, *FFU* final follow-up, *Post-op RNP* postoperative radial nerve paralysis, *LCP* locking compression plate, *MDLS* multi-directional locking screw system, *NA* not applicable

**Table 2:** ORIF group

Case	Age (years)	Gender	Side	AO/OTA classification	Plate type	Incision length (cm)	Bleeding volume(ml)	Fluoroscopy times	operative time (min)	HS (days)	BH (weeks)	FFU (months)	Post-op RNP	QuickDASH score	Constant score
1	56	Male	Right	A3.2	LCP	14	170	5	150	10	16	19	NA	6.8	74
2	24	Female	Right	B1.3	Dual LCP	18	150	10	180	9	18	24	NA	2.3	95
3	20	Female	Left	B1.3	LCP	13	145	9	146	9	18	22	NA	4.5	90
4	19	Female	Right	B1.3	Dual LCP	19	110	11	190	10	19	15	NA	4.6	90
5	17	Female	Left	B1.3	LCP	13	135	8	144	10	15	21	NA	0.5	95
6	41	Female	Left	A3.2	LCP	14	130	10	135	9	18	16	NA	2.3	95
7	31	Female	Left	A3.2	LCP	15	145	8	145	11	15	12	NA	2.3	90
8	16	Male	Left	A3.3	LCP	13	160	8	135	9	16	24	NA	4.2	85
9	23	Male	Left	A3.2	LCP	12	150	6	130	10	15	23	NA	2.3	83
10	45	Female	Right	A1.2	LCP	15	150	7	140	9	18	25	NA	15.9	59
11	39	Male	Left	A2.2	LCP	16	145	6	145	9	16	20	NA	4.6	85
12	25	Female	Right	B1.3	Dual LCP	20	150	12	178	10	18	14	NA	4.5	89
13	29	Male	Left	A1.3	Dual LCP	19	110	11	185	11	18	18	NA	4.0	90
14	73	Female	Right	A1.3	LCP	12	140	8	150	13	17	12	NA	2.4	74

*BH* bone healing, *FFU* final follow-up, *Post-op RNP* postoperative radial nerve paralysis, *LSTP* locking straight tibial plate, *LSFP* locking straight femoral plate, *NA* not applicable, *RSD* reflex sympathetic dystrophy, *HSD* hospital stay (days)

### Statistical analysis

In our study SPSS 25.0 was used to analyze the data. Data were grouped into groups. Measurement data were expressed as ($\overline{x}$±s), and an independent sample t test was adopted (*P* < 0.05 was considered statistically significant) (Table 3).

**Table 3:** Comparison of related indicators

Group	Number	Age (years)	Incision length (cm)	Bleeding volume (ml)	Fluoroscopy (times)	operative time (min)	HSD (days)	BH (weeks)	FFU (months)	QuickDASH score	Constant score
MIPO group	14	47.79 ± 18.61	7.79 ± 2.39	96.07 ± 14.96	20.07 ± 3.22	110.57 ± 21.90	6.29 ± 1.49	14.94 ± 0.99	16.50 ± 10.60	2.53 ± 1.34	88.57 ± 5.60
ORIF group	14	32.71 ± 16.51	15.21 ± 2.75	142.14 ± 16.72	8.5 ± 2.10	153.79 ± 20.31	9.93 ± 1.14	16.93 ± 1.38	18.14 ± 6.38	4.34 ± 3.67	85.29 ± 10.14
t value	–	1.914	− 7.206	− 7.295	11.054	− 5.426	− 12.599	− 3.925	− 0.449	− 1.944	1.097
P value	–	0.078	0.000	0.000	0.000	0.000	0.000	0.002	0.661	0.074	0.293

*HS* days of hospitalization, *BH* bone healing, *FFU* final follow-up, *HSD* hospital stay (days)

## Results

Tables 1 and 2 respectively summarize the results and characteristics of the MIPO group and the ORIF group. All patients were free of radial nerve palsy before and after surgery. Compared to the ORIF group, the incision length (7.79 ± 2.39 cm), was shorter, intraoperative blood loss (96.07 ± 14.96 mL) was less, and the operation time (110.57 ± 21.90 min), hospital stay (6.29 ± 1.49 days) and fracture healing time (14.94 ± 0.99 weeks) were all significantly shorter in the MIPO group (*P* < 0.05) (Table 3). The number of intraoperative fluoroscopy images (20.07 ± 3.22) was significantly higher in the MIPO group (*P* < 0.05). There were no significant differences in age (*P* = 0.078), QuickDASH score (*P* = 0.074) or Constant score (*P* = 0.293) between the two groups and no postoperative complications occurred in any of the patients (Table 3).

## Discussion

Although ORIF is the main surgical method for the treatment of humeral shaft fracture, the exposure of the fracture site by open reduction damages the blood supply of the humerus, which may affect fracture healing. The rate of fracture nonunion reported in the literature is 6–15% [11]. The traditional anterolateral approach may cause iatrogenic injury to the radial nerve, and iatrogenic radial nerve paralysis occurs in 0–12% of cases [11]. Extensive intraoperative exposure of soft tissue in ORIF also increases the incidence of deep postoperative infection of the incision [12]. In recent years, scholars have applied MIPO technology in the treatment of humeral shaft fracture and achieved good results. The MIPO technique uses small incisions far away from the fracture site to avoid direct exposure to the fracture, theoretically improving the healing rate and reducing the risk of infection through the incision [13]. An LCP is mostly used in a MIPO operation, which does not need to be completely fitted to the bone surface [14, 15]. Use of a locking screw reduces the pressure of the plate on the bone, protects the periosteal blood supply, and is conducive to fracture healing. We compared the MIPO and ORIF operative techniques, and found that the MIPO group required a shorter incision length, suffered less blood loss, and had a shorter postoperative hospitalization time and shorter fracture healing time, but on the other hand this technique involved an increase in the amount of radiation exposure during the operation, leading to a certain amount of radiation damage to physicians and patients. In terms of operation time, fracture healing time, and postoperative complications, the two groups showed no significant differences [16].

Regarding postoperative recovery, according to the results of this study, the MIPO group was significantly better than the ORIF group, with a markedly shortened postoperative recovery time [17]. Our results showed that MIPO can restore limb length, correct deformity, restore the axis angle, requires a smaller incision, and leaves smaller and less disfiguring scars. MIPO conforms to the principle of biological treatment of fracture, promotes stability and reconstruction of the local blood supply, reduces the incidences of infection or delayed union, and promotes recovery of patients’ shoulder joint function. In this retrospective study, all patients had healed fractures, perhaps because of the small sample size.

The aim of our study was to validate the efficacy and safety of the MIPO anteromedial approach for the treatment of middle and distal humeral shaft fractures by combining the advantages of the anteromedial approach and the MIPO technique. Anatomically, the anteromedial approach to MIPO is a safe and effective approach for the treatment of middle–distal humeral shaft fractures [18]. The pronator teres and brachialis muscles were pulled laterally, protecting the median nerve and brachial artery. The mean distance from the distal incision to the median nerve was 2.34 cm (95% CI, 2.18–2.50 cm) [19]. Radial nerve palsy is known to be a major complication of the anterior and anterolateral MIPO technique, and the incidence of radial nerve palsy with the posterior MIPO technique is 5.4% [20]. The lateral approach to the distal humerus in MIPO inevitably affects the radial nerve, while the anteromedial approach avoids the risk of radial nerve injury.

**Fig. 4.**
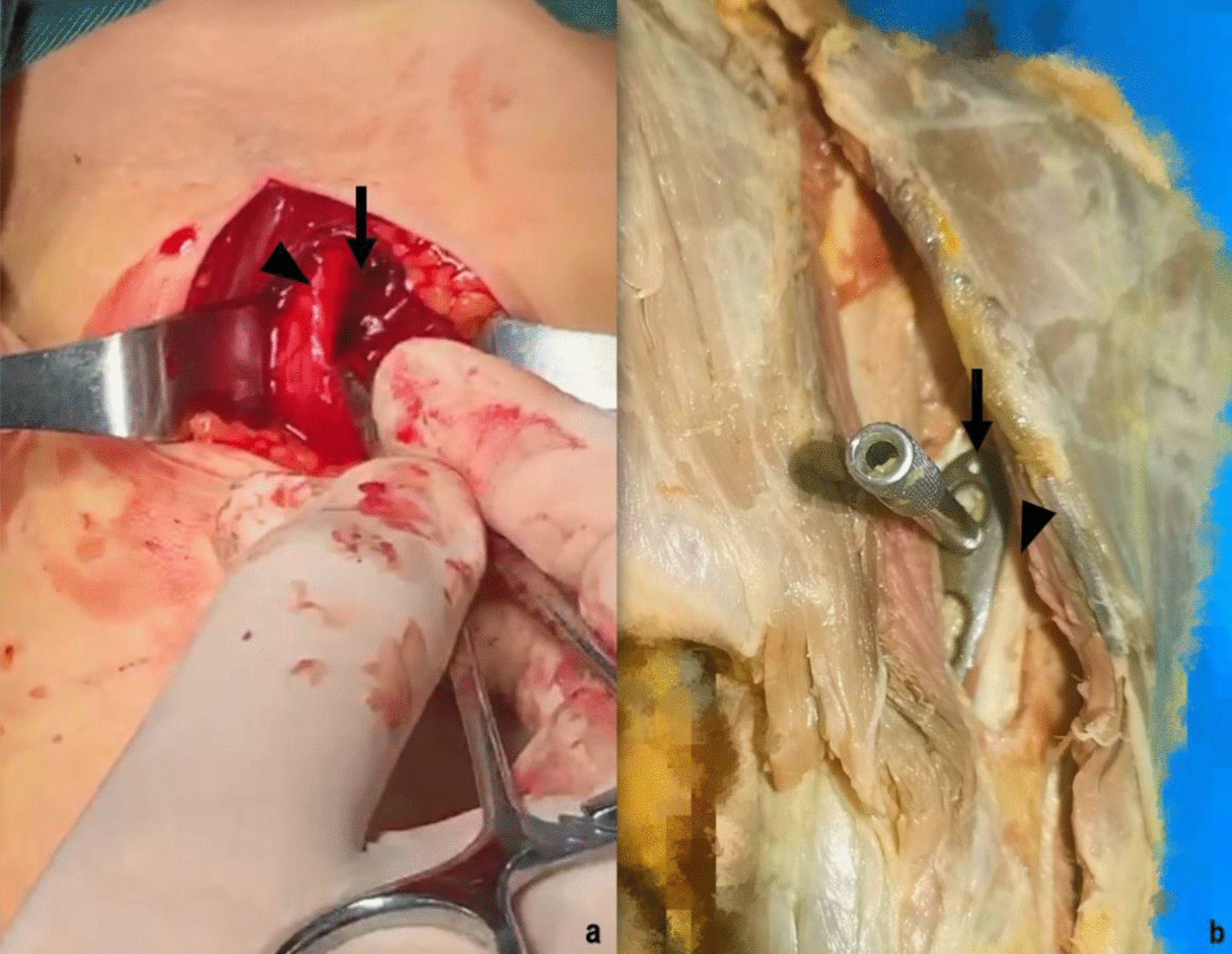
**a** Intra-operative photograph showing the proximal incision, below the (long) head of the biceps brachii (triangular arrow head), insertion of the plate position (slim arrow). **b** A cadaver study proximal incision, the (long) head of the biceps brachii (triangular arrow head) was pulled laterally and subcutaneous MIPO tunnels created (slim arrow)

Ulnar nerve injury is also a concern with the anteromedial approach to MIPO of the distal humerus [21], as the ulnar nerve runs near the apex of the epicondyle within the humerus, and the distal plate is located lateral to the ulnar nerve in the treatment of a fracture in the middle and lower part of the humerus. The distal screw is very close to the ulnar nerve and the space available for the plate is narrow. In our experience, in distal humerus fractures, we prefer to use a multi-directional locking screw system (MDLS) for distal locking screw monocortical fixation, and if necessary, a plate can be added laterally to stiffen the fixation [19] A study by Cañada-Oya et al. [19] concluded that a proximal plate may affect the long head tendon of the biceps brachii. Based on our clinical experience, a proximal plate pulls the biceps tendon medially to the patient but the plate can be placed on the deltoid insertion and part of the deltoid insertion can be removed if necessary. According to the long-term patient follow-up, if the plate was located below the long head bond of the biceps, there was no discomfort associated with movement of the shoulder joint, so it was not necessary to choose a shorter plate. If the plate is short and is located below the belly of the biceps brachii, proximal screw fixation will be difficult due to the greater soft tissue coverage [19]. (Fig. 4).

The advantages of this new anteromedial minimally-invasive approach include the ability to place the external fixator on the lateral side of the humerus during the operation to maintain intraoperative reduction without compromising the operation [19]. In clinical practice, we prefer to use a lag screw to pull the humerus proximally to the plate and reduce the fracture with the plate. In cases of complex fractures of the distal humerus, we can use an anterolateral approach to assist plate fixation [22, 23].

Based on our study, the anteromedial MIPO approach may be an alternative for middle and distal humerus fractures. If the fracture extends distally and the fixation is unstable, we recommend a lateral approach to assist fixation by the MIPO technique [24, 25]. This approach may also increase the stability of fixation, especially in cases of severe osteoporosis, periprosthetic fractures, and pathological fractures requiring biplanar fixation. Biomechanical studies of the human skeleton have shown that anteromedial plates provide better stability than anterolateral or posterolateral plates in the treatment of mid-humeral fractures, and that the anteromedial minimally-invasive approach is not suitable for the treatment of proximal humeral fractures due to the lack of adequate fixation sites [26]. A dual plate can be used in combination with an anterolateral or lateral approach to reconstruct the medial and lateral columns of the distal humerus while preserving blood supply to the surrounding soft tissues and hastening fracture healing [9].

## Conclusions

Based on our clinical practice studies, the anteromedial approach to MIPO allows exposure of the proximal and distal incisions without exposing the nerves and vessels. However, it is difficult to insert a screw between the distal and proximal incisions. This method can be used as an option for extra-articular fractures of the middle and distal humerus shaft with less trauma and is a safe and feasible surgical method. When presenting a novel technique, even a rather small case series might be relevant, so more medical records and long-term follow-up studies are still needed to further verify this conclusion.

## Data Availability

The data and materials during the current study are available from the corresponding author on reasonable request.

## Abbreviations

MIPO: Minimally-invasive plate osteosynthesis
ORIF: Open reduction and internal fixation
LCP: Locking compression plate
MDLS: Locking compression plate multi-directional locking screw system
OTA: Orthopaedic Trauma Association
